# IL13RA2-integrated genetically engineered mouse model allows for CAR T cells targeting pediatric high-grade gliomas

**DOI:** 10.21203/rs.3.rs-5398280/v1

**Published:** 2024-12-11

**Authors:** Maggie Seblani, Markella Zannikou, Joseph Duffy, Rebecca Levine, Aditi Thakur, Montserrat Puigdelloses-Vallcorba, Crag Horbinski, Jason Miska, Dolores Hambardzumyan, Oren Becher, Irina Balyasnikova

**Affiliations:** Lurie Children’s Hospital; Northwestern University; Northwestern University; Northwestern University; Northwestern University; Icahn School of Medicine at Mount Sinai; Northwestern University; Northwestern University; Icahn School of Medicine at Mount Sinai; Icahn School of Medicine at Mount Sinai; Northwestern University

## Abstract

Pediatric high-grade gliomas (pHGG) and pediatric diffuse midline gliomas (pDMG) are devastating diseases without durable and curative options. Although targeted immunotherapy has shown promise, the field lacks immunocompetent animal models to study these processes in detail. To achieve this, we developed a fully immunocompetent, genetically engineered mouse model (GEMM) for pDMG and pHGG that incorporates the glioma-associated antigen, interleukin 13 receptor alpha 2 (IL13RA2). Utilizing the RCAS-Tva delivery system in Nestin-Tva mice, we induced gliomagenesis by overexpressing PDGFB and deleting p53 (p53^fl/fl^) or both p53 and PTEN (p53^fl/fl^ PTEN^fl/fl^), with or without IL13RA2 in neonatal mice. *De novo* tumors developed in models with and without IL13RA2, showing no statistical difference in onset (n = 33, 38 days, p = 0.62). The p53^fl/fl^ PTEN^fl/fl^ tumors displayed more aggressive characteristics (n = 12, 31 days). Tumors exhibited features typical of high-grade glioma, including infiltration, pseudopalisading necrosis, and microvascular proliferation. They also showed a high Ki-67 index, variable IL13RA2 expression, a high frequency of CD11b + macrophages, and a low proportion of CD3 + T cells. The model proved effective for evaluating IL13RA2-targeted immunotherapies, with a significant response to CAR T-cell treatment that extended survival (46 days vs. 28 days control; p < 0.0001) and achieved 25% long-term survival in mice. This model facilitates the preclinical assessment of IL13RA2-directed therapies and holds potential for clinical application.

## INTRODUCTION

Among pediatric cancers, diffuse midline gliomas (DMG) are notorious for their universally poor outcomes^1^. The prototypical diffuse midline glioma, diffuse intrinsic pontine glioma (DIPG), is an aggressive tumor that has a median survival of less than one year from diagnosis and is currently incurable^2^. Radiation provides limited symptom control and extends overall survival on average by only three months. The lack of sustained response to conventional therapies reinforces the pressing need for new therapies.^3,4^

The recent success of cancer immunotherapy in adults and children with hematologic malignancies has intensified efforts to develop immunomodulatory applications in central nervous system (CNS) tumors. Advances in molecular characterization of midline gliomas via increased tissue sampling at diagnosis and post-mortem have enabled the subgrouping of tumors and identified molecular drivers^1,5–7^. Surface markers, such as GD2 and B7-H3, have previously been characterized based on expression levels and degree of inter- and intra-patient heterogeneity^8,9^. Preclinical studies of chimeric antigen receptor-modified T-cells (CAR T-cells) targeting GD2 and B7-H3 (CD276) have translated into clinical trials (NCT04196413, NCT04099797, NCT04185038)^10,11^. Interleukin-13 receptor alpha 2 (IL13RA2) is another attractive immunotherapeutic target in pediatric HGG (pHGG). High expression of IL13RA2 has been described in DIPG and other pHGG tissues^4,12,13^. Within adult-type diffuse gliomas, IL13RA2 targeting therapies have been the focus of multiple preclinical and clinical studies. One study utilized the IL13RA2 epitope for a glioma vaccine^14^, and a phase I clinical trial featuring IL13RA2 targeting CAR T-cells in IL13RA2-positive glioblastoma in adults has been recently completed^15^. Autologous CAR T-cells targeting IL13RA2 in patients with recurrent or refractory pHGG are currently being evaluated (NCT04510051). Overall, the advancements in molecular characterization, cellular engineering capabilities, and the growing number of clinical trials justify the investigation of targeted immunotherapy for DMG.

Orthotopic implantation of murine glioma cell lines into immunocompetent hosts is predominantly used for preclinical evaluation of targeted immunotherapies. However, the inability of these models to recapitulate the tumor microenvironment and failure to express targetable human antigens remains a limitation for clinical translation. Therefore, developing an immunocompetent preclinical model which both recapitulates DMG and expresses targetable human antigens is paramount. Although these advances provide a growing number of targets for immunotherapy and justify further investigation into IL13RA2 as a target, the lack of an immunocompetent preclinical model of DIPG that expresses human IL13RA2 hinders the development of IL13RA2-directed immunotherapies.

A genetically engineered mouse model (GEMM) is one such system that permits tumorigenesis in the presence of a competent immune system^13,16^. GEMMs enable the spatiotemporal introduction of specific genetic abnormalities and permit the investigation of various stages of tumor development, including tumor initiation^17^. The RCAS/Tva system is a powerful genetic engineering tool that enables specific somatic cell gene transfer into cells engineered to express the Tva receptor^18^. Holland and colleagues previously developed GEMM, which uses the RCAS/Tva system to delete p53 in Tva-positive cells and induce gliomagenesis^18^. Using engineered mice with Tva receptors in nestin-positive progenitor cells, a candidate cell of origin for DIPG^19,20^, Becher and colleagues expanded this model to induce brainstem gliomogenesis in newborn mice^21^. The strength of this model is in its versatility, as it enables customization to express exogenous human genes of interest, namely immunotherapeutic targets, within Tva-expressing cells.

We have previously developed an IL13RA2-specific antibody and incorporated it into CAR T-cells and bi-specific T-cell engagers for preclinical evaluation in adult glioblastoma^22,23^. Here, we integrated IL13RA2 into a construct encoding for PDGFB and used the RCAS/Tva system to develop a novel immunocompetent GEMM that both recapitulates diffuse midline gliomas and expresses the human glioma-associated antigen IL13RA2. We tested the feasibility of this model to assess the anti-tumor efficacy of our IL13RA2-CAR T-cells. Engineering diffuse midline and hemispheric high-grade gliomas that express IL13RA2 will fill a significant gap in existing models and allow preclinical mechanistic and therapeutic studies in corresponding pediatric brain tumors.

## MATERIALS and METHODS

### Analysis of TCGA patient data sets

Levels of IL13RA2 gene expression in pediatric brain tumors were assessed using a publicly available microarray expression profile Gump database (tumor samples: 106, non-tumor: 6; GEO ID: GSE68015). RNA-seq data from Gump were visualized using GlioVis (http://gliovis.bioinfo.cnio.es/), with the relative expression of IL13RA2 determined from log2 transformation of normalized count reads.^24^ Statistical significance between pediatric tumors was determined using Tukey’s Honest Significant Difference (HSD) within GlioVis, with one asterisk (*) where p < 0.05, two (**) where p < 0.01, and three (***) where p <0.001.

### Histology and immunohistochemistry of human samples

Human tumor samples were initially obtained under Institutional Review Board approved protocol (#IRB 2019–3164) per Ann and Robert H. Lurie Children’s Hospital of Chicago. All samples were de-identified. Tissue sections were stained with H&E and for IL13RA2 (R&D antibody, cat AF146) IHC. Scoring was performed independently by a neuropathologist (C.H.). Scores were established on the percent positively labeled tumor cells; 0 is no positivity, 1 is ≤10% positive tumor cells, 2 is 10–50% positive tumor cells, 3 is ≥ 50% positive tumor cells. PTEN immunohistochemistry staining was performed on a Leica Bond Rx platform (Leica). Primary anti-PTEN antibodies were diluted 1:100 (Cell Signaling, #9559). Anti-rabbit secondary antibodies were purchased from Leica. Digital slide images were acquired using a Nanozoomer 2.0HT whole-slide scanner (Hamamatsu Photonic K.K.) and observed offline with NDP.view2 software (Hamamatsu).

### Cell lines and cell culture reagents

DF-1 (chicken fibroblast) cell line was obtained from ATCC (cat #CRL-12203). All cell lines were cultured in Dulbecco’s Modified Eagle’s Medium (DMEM) (ATCC, Manassas, Virginia; Cat #30–2002) supplemented with 10% fetal bovine serum (FBS) (R&D Systems, Minneapolis, MN), 1% penicillin/streptomycin (P/S) (Corning, Corning, NY; Cat #30–002-CI) and 2mM L-glutamine (Corning, Corning, NY; Cat # 25–005-CI). Dulbecco’s Phosphate-buffered saline (PBS) was obtained from Thermo Fisher. GeneJuice was purchased from (Millipore Sigma).

### Cloning IL13RA2 in vector encoding for PDGFB cDNA

The cDNA encoding T2A-IL13RA2 was synthesized by GenScript, subcloned into the RCAS-PDGFB-HA vector^25^ following the PDGFB cDNA using CloneEZ method, and verified by Sanger sequencing (GenScript, Piscataway, NJ). The plasmid was amplified in DH5a cells (NewEngland Biolabs, Ipswich, MA; Cat #C2987H) and purified using a plasmid purification midi-kit (Qiagen, Hilden, Germany; Cat # 12145).

### Generation of DF-1 cells producing virus encoding for PDGFB, RCAS-Cre, and PDGFB + IL13RA2

Our approach expands upon a previously described use of the RCAS-Tva system for GEMM creation.^14^ Briefly, we cultured DF-1 cells in optimal growth conditions in DMEM supplemented with 10% FBS, 1% P/S, and 2 mM L-glutamine. Cells were transfected with RCAS plasmids (RCAS-Cre, PDGFB, and PDGFB + IL13Rα) using GeneJuice (Millipore Sigma, Burlington, MA; Cat # 70967), per the manufacturer’s protocol. To propagate the necessary cell number and adequate retrovirus for intracranial injection, we serially passaged the three transfected cell groups upon reaching 80–90% density for up to five passages.

#### Generation of CAR T-cells for targeting of IL13RA2-expressing de novo tumors.

Murine CAR T-cells targeting IL13RA2 were generated as we previously reported^26^. CAR T-cells were tested for their ability to kill newly generated cell lines in co-culture with newly generated IL13RA2-expressing glioma lines for 48 hours at 1:20 target-to-effector (T:E) ratio.

### Western blot

Cells were cultured in complete DMEM as previously described. Cells were collected, pelleted, and then resuspended into one mL of cold Dulbecco’s Phosphate-buffered saline (PBS) (4°C). Cells were counted via the trypan-blue exclusion method, pelleted by centrifugation (400x g, 5 min), and lysed in ice-cold Mammalian Protein Extraction Reagent (mPER) (Thermo Scientific, Waltham, MC) at a concentration of 20,000 cells/μL mPER. Protein concentration was measured using a Pierce^™^ bicinchoninic acid (BCA) protein assay kit (Thermo Scientific. Protein samples were then prepared in Laemmli Sample Buffer (Bio-Rad, Hercules, CA; Cat #1610747) with 2.5% 2-Mercaptoethanol (Sigma Aldrich, St. Louis, MO; Cat #M6250) and denatured at 95°C for 5 min. Protein samples and 250 kDa Precision Plus Protein standard (Bio-Rad Laboratories, Hercules, CA) were loaded into each well of a 4–20% Midi-PROTEAN^®^ TGX Stain- Free^™^ gel (Bio-Rad). Proteins were separated by size using SDS-polyacrylamide gel electrophoresis (SDS-PAGE) and transferred onto a methanol-activated 0.2 μm PVDF membrane (Millipore Sigma, Burlington, MA; Cat #IPVH08100) using the Trans-Blot^®^ Turbo Transfer System^™^ (Bio-Rad). Membranes were blocked with 5% non-fat dry milk (NFDM) in tris-buffered saline plus 0.1% Tween-20 (TBST) (Boston Bioproducts, Ashland, MA; Cat #IBB180) for 2 hours at RT. Membranes were incubated with anti-IL13RA2 monoclonal antibody (1:1000) (R&D Systems, AF146), anti-HA tag antibody to detect PDGFB (1:1000) (Cell Signaling Technology, 3724), and anti-glyceraldehyde 3-phosphate dehydrogenase (GAPDH) Monoclonal Antibody (1:1000) (Cell Signaling Technology, 2118) in 5% NFDM-TBST overnight at 4°C. The next day, membranes were washed with TBST and incubated with anti-rabbit HRP-linked Secondary Antibody (1:1000) (Cell Signaling Technology, 7074) for GAPDH and anti-HA tag and chicken anti-goat Secondary Antibody (Santa Cruz Biotechnology, sc-2953) for IL13RA2. Blots were washed with TBST, incubated with Clarity^™^ Western ECL Substrate (Bio-Rad), and detected via chemiluminescent image acquired by ChemiDoc^™^ MP Imaging System (Bio-Rad).

### Flow Cytometry

Cells were maintained in culture with complete DMEM. Cells were collected, pelleted, and incubated with TruStain Fc Blocking Antibody (1:200 in 1% FBS in DPBS) (4°C). Next, 100,000 cells were added to each tissue culture-treated V-bottom plate well. Cells were pelleted and stained on ice with PE anti-human IL13RA2 (Biolegend, 354403). Live cells were labeled with a fixable viability stain (Invitrogen, 65–0865-14). Cells were then fixed, permeabilized, and stained with Alexa-Fluor 488 Anti-HA antibody (Biolegend, 901509) to detect PDGFB. Antibodies against CD45, CD4, CD8, CD69, CD25, Granzyme B, CD44, FoxP3, CD11b, Ly6C and Ly6G (BioLegend) were used for analysis of T-cell activation and myeloid cell presence in tumors environment in response to CAR T-cell treatment. Data acquisition was done using the FACSymphony A5-Laser Analyzer, BD.

### Histology and immunohistochemistry of mouse samples

At selected time points or based upon clinical deterioration, animals were perfused with PBS to collect brain tissue for sectioning. Tissue was fixed in 4% paraformaldehyde (PFA), then paraffin-embedded, cut into 4-micron sections, and stained with hematoxylin and eosin (H&E). Further immunohistochemistry staining was obtained using following antibodies: IL13RA2 (R&D AF146; 1:200), Olig2 (Abcam ab109186; 1:2000), Ki67 (Abcam ab16667; 1:500), CD3 (Abcam ab16669; 1:1000), CD11b (Abcam ab133357; 1:3000). To demonstrate the PTEN and H3K27me3 absence in glioma cells, the staining of tissues was carried out using PTEN (138G6) and Tri-Methyl-Histone H3 (Lys27) rabbit (C36B11, 1:300) antibodies recognizing wild-type PTEN and H3.3 histon proteins (Cell Signaling).

### Animal Studies

Nestin-tva (Ntv-a) p53^fl/fl^ or Ntv-a p53^fl/fl^ PTEN ^fl/fl^ (obtained from Dr. Becher Laboratory) mice were bred in-house per animal protocols approved by the Northwestern University Institutional Animal Care and Use Committee (IACUC). Our study included male and female mice with no effect of sex as a biological variable being evaluated explicitly in the study. Suspensions of virus-producing cells were injected into the hindbrain of Ntv-a p53^fl/fl^ mice pups between three to five post-natal days of age, using cryoanesthesia during the procedure. We evaluated two experimental groups: 1) RCAS-PDGFB and 2) RCAS-PDGFB + IL13RA2 with p53 or p53/PTEN loss, with the former as the established model and the latter our new model with a combined cell count of 1–2×10^5^ cells/mL per pup (a 1:1 ratio of RCAS-Cre with PDGFB or PDGFB + IL13RA2) suspended in PBS for a total volume of 1.2 μL. In a separate experiment, cells producing RCAS-H3.3 K27M were added to Ntv-a p53^fl/fl^ PTEN ^fl/fl^ model. To generate DIPG-bearing mice, the injection site was approximately 2 mm posterior to the bregma along the midline using a Hamilton syringe and custom needle. Hemispheric tumors were induced by injecting pups about 1 mm posterior to the bregma and 1 mm right of the midline. Upon postprocedural recovery and rewarming, the pups were returned to their nursing mother. Following injection, mice were monitored closely for symptoms consistent with tumor involvement, including ataxia, seizures, enlarged head, and weight loss. For assessment with CAR T-cell therapy, mice bearing *de novo* cortical tumors were injected with 1 ×10^6^ of non-transduced control or CAR T-cells in the same coordinates as an original tumor initiation site approximately two weeks after tumor induction. Brains were collected 72 hours post CAR T-cells injection for flow cytometric analysis of infiltrating immune cells. Survival outcomes were monitored, and mice were designated as long-term survivors (LTS) if they demonstrated no signs of disease at a 120-day post-injection interval. Brains of LTS mice were collected and histologically evaluated to confirm tumor absence.

### Generation of cell lines for orthotopic model

Cell lines were generated by processing tumor-bearing tissue obtained from symptomatic mice. Mice were anesthetized with a solution consisting of 200 mg/kg ketamine and 20 mg/kg xylazine and perfused with PBS before neoplastic tissue was processed with the NeuroCult^™^ enzymatic dissociation Kit (StemCell Technologies, 05715) per manufacturer’s instructions. Cells were maintained as neurospheres in NeuroCult^™^ proliferation media (Stem Cell Technologies, Vancouver, Canada; Cat #05072) with the addition of mouse cell proliferation supplement (Stem Cell Technologies, Cat #05701), P/S, heparin at 2μg/mL and 10ng/mL epidermal growth factor, and 20ng/mL basic fibroblast growth factor. For orthotopic experiments, mouse pups were injected with 1 × 10^5^ cells (1.2 μL) per animal in the hindbrain and the right cortex, as described in animal studies.

### Statistics

Statistical data analyses were executed using GraphPad 8 (Prism, La Jolla, CA) and Microsoft Excel. Significance was defined as *p* less than 0.05 in all statistical tests. P values were established as follows: *p < .05, **p < .01, *** *p* < .001, and **** *p* < .0001. As indicated, data in two groups were analyzed for statistical significance using the unpaired Mann-Whitney test or an unpaired two-tailed Student’s t-test. One- or two-way ANOVA was used for multiple groups, followed by Tukey’s or Dunnett’s multiple comparisons test. Animal survival analysis was performed by generating Kaplan-Meier plots using the Wald test to determine the p-value.

## RESULTS

### Analysis of IL13RA2 expression in patient tissues

**Figures 1:**
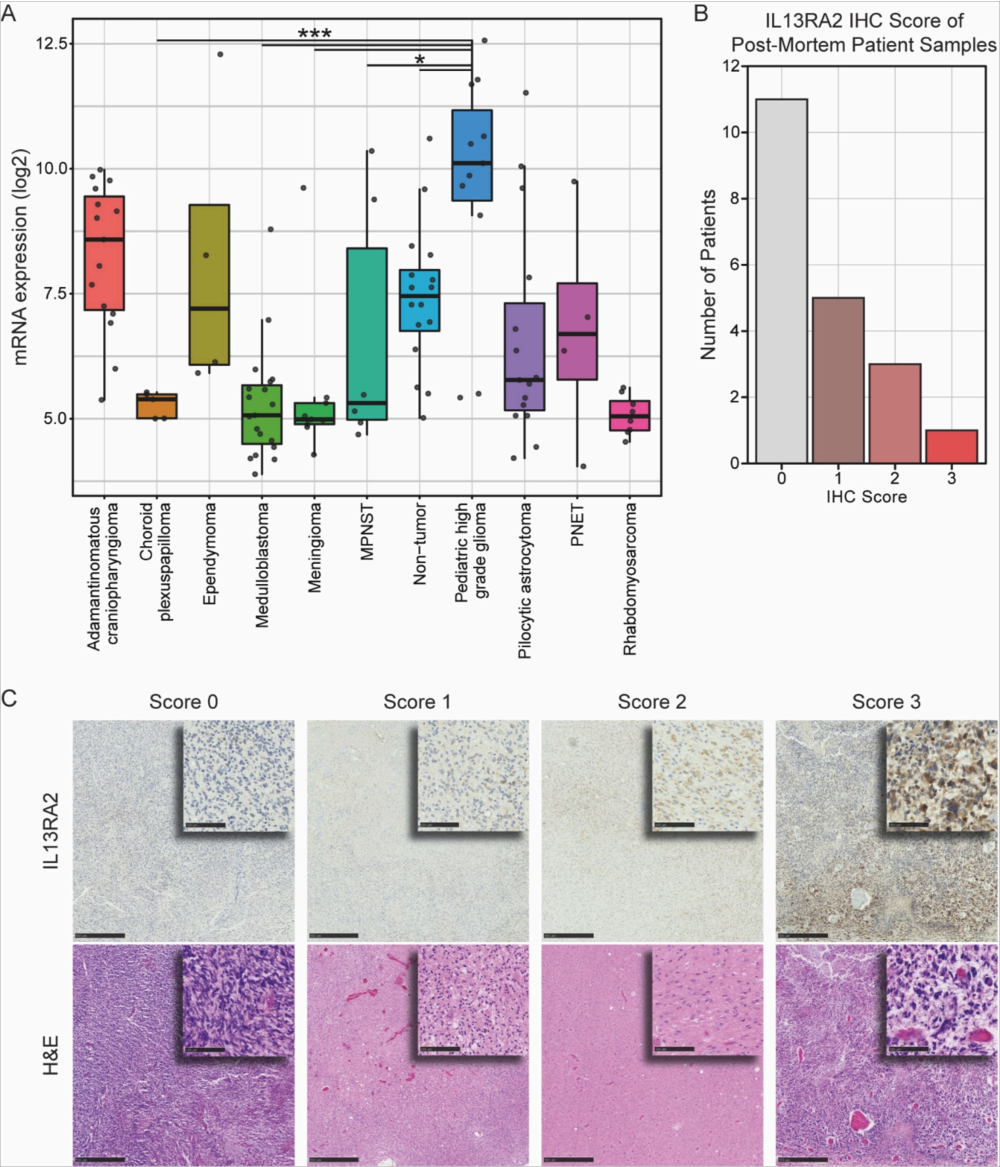
IL13RA2 shows heterogenous expression in human DIPG. (A) IL13RA2 gene expression in pediatric brain tumors from Gump database was analyzed using GlioVis (http://gliovis.bioinfo.cnio.es/). Tukey’s Honest Significant Difference (HSD) was performed to compare IL13RA2 gene expression between pediatric tumors. Significance was denoted in the graph by one asterisk (*) where p<0.05, two (**) where p<0.01, and three (***) where p<0.001. (B) IL13RA2 histopathological scores of post-mortem patient samples. (C) Representative images of each IL13RA2 histopathological score in post-mortem human pDMG patient samples (top panel) and corresponding H&E stains (bottom panel). Each image includes high-power insets at low-power fields (top right hovering image)

IL13RA2 expression in adult-type diffuse high-grade gliomas is well established, and it has been used as an immunotherapeutic target in multiple preclinical and clinical studies.^22,23,27,28^ To determine the relative expression in pediatric CNS tumors, we used GlioVis to visualize IL13RA2 gene expression within the publicly available Gump database (GEO ID: GSE68015). Increased IL13RA2 mRNA expression was found in pediatric-type diffuse high-grade gliomas (pHGG) compared to non-neoplastic brain tissue and other pediatric brain tumors (Fig. 1A). Furthermore, elevated IL13RA2 gene expression was seen in pHGG compared to low-grade CNS tumors (Fig. 1A). To validate these findings further, we assessed tissue expression of IL13RA2 in twenty post-mortem pediatric DIPG samples using immunohistochemistry (IHC). Nine of the twenty (45%) samples were positive for IL13RA2, receiving histopathological scores of 1 (n = 5), 2 (n = 3), and 3 (n = 1) (Fig. 1 B&C).

### Generation and characterization of the RCAS-Tva model of high-grade pediatric diffuse glioma expressing IL13RA2

**Figure 2:**
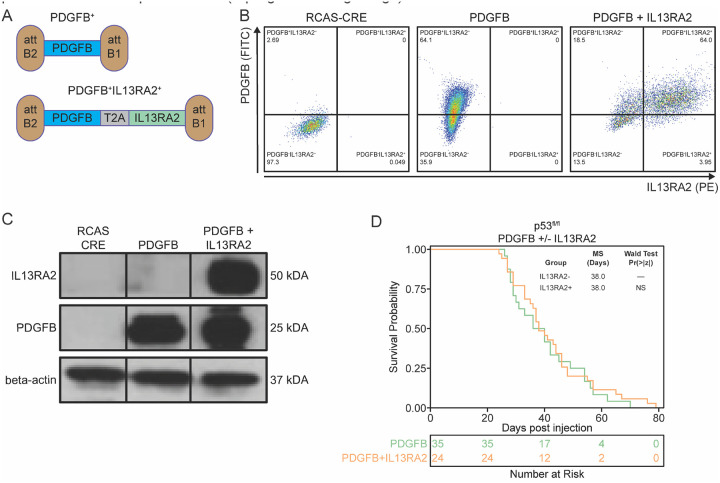
Analysis of GEM models of midline glioma expressing IL13RA2. (A) Visual schematic of RCAS vector generated with PDGFB and IL13RA2 transgenes flanked by recombination att sites used to transfect DF-1 cells. Validation of PDGFB and IL13RA2 expression in DF-1 by (B) flow cytometry and (C) western blot. (D) Comparison of survival for Nestin-Tva;p53^fl/fl^ mice injected at midline location with DF-1 producing virus containing RCAS-CRE and PDGFB or PDGFB+IL13RA2.

To generate *de novo* tumors, we first cloned human IL13RA2 in a PDGFB construct using a T2A cleaving peptide. Next, we transfected DF-1 cells with either RCAS-Cre, RCAS-PDGFB, or RCAS-PDGFB + IL13RA2 constructs. Subsequent flow cytometry (FSC) analysis of DF-1 cells showed robust expression of PDGFB (64.1%) and PDGFB + IL13RA2 (64.0%) (Fig. 2B). Secondary confirmation of successful RCAS-PDGFB and RCAS-PDGFB + IL13RA2 transfection was achieved via western blotting for PDGFB and IL13RA2 (Fig. 2C). Following transduction confirmation, we evaluated growth dynamics of de novo tumors *in vivo* by infecting nestin-expressing progenitors in either the midbrain or right cortex of 4–5-day-old Nestin-tva p53^fl/fl^ mice to model pDMG and pHGG, respectively. No significant changes were noted in the median survival of mice between RCAS-PDGFB (n = 35, 38 days) and RCAS-PDGFB + IL13RA2 (n = 35, 38 days) of pDMG models (Fig. 2D).

#### Histopathological analysis of de novo tumors, generation of cell lines, and orthotopic transplantation

**Figure 3:**
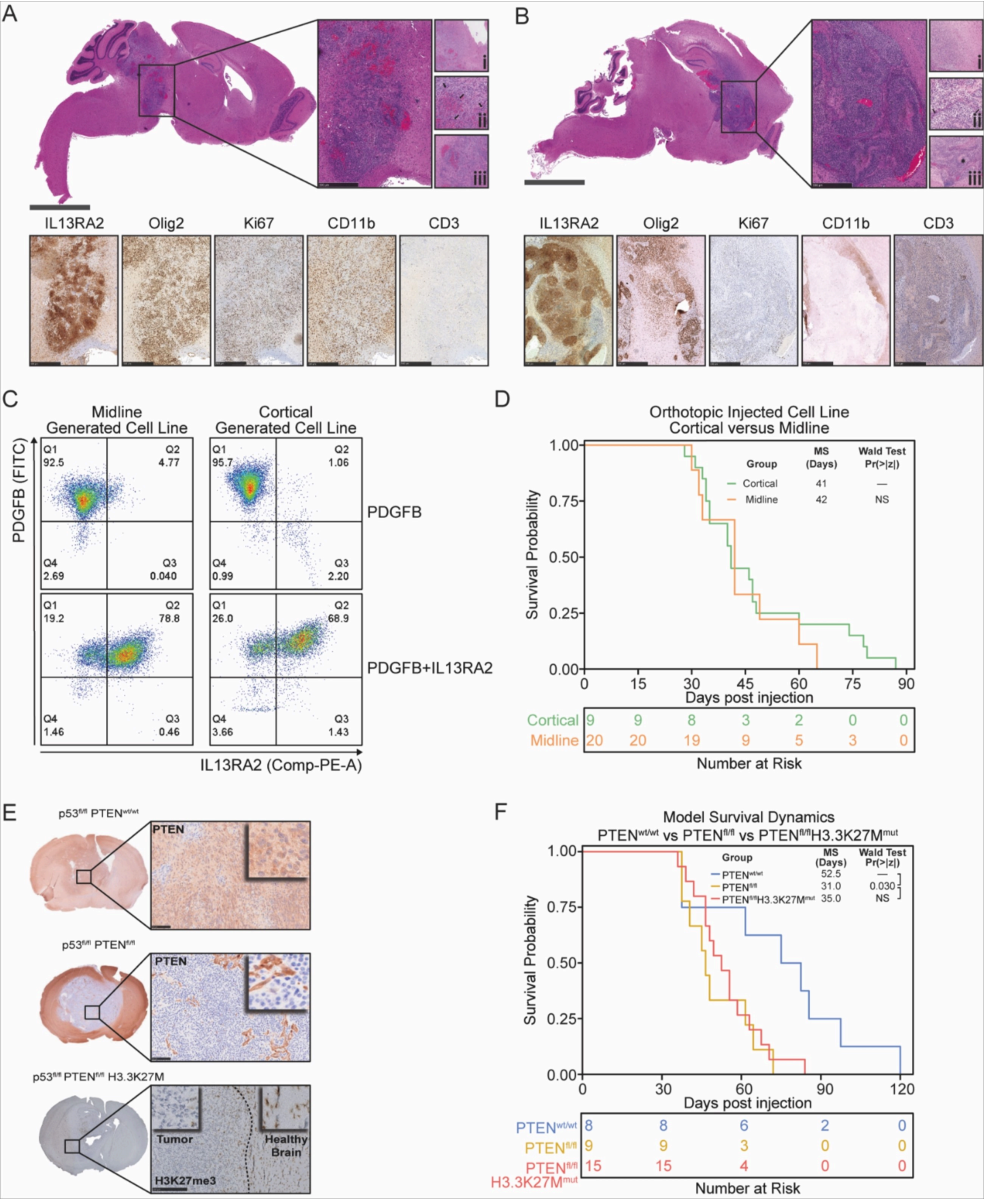
Histopathological analysis of *de novo* tumors. *de novo* tumor generation for (A) diffuse midline glioma through midline injection, 49 days post-injection, and (B) supratentorial high-grade glioma through cortical injection, 50 days post-injection. (A-B) Whole brain tissue section and tumor stained with H&E. Higher magnification of inset images reveal (i) infiltrative tumor cells, (ii, arrows) microvascular proliferation, and (iii, asterisk) pseudo-palisading necrosis. (C). Flow cytometric analysis of IL13RA2 and PDGFB expression in cell lines generated from the midline and cortical *de novo* tumors. (D) Survival analysis of orthotopically injected cell lines in the midline and cortical location of Nestin-Tva; p53^fl/fl^ mice. (E) Histological validation of PTEN expression, PTEN loss, and H3K27me3 loss in *de novo* tumors derived from p53^fl/fl^/PTEN^wt/wt^, p53^fl/fl^/PTEN^fl/fl^, and p53^fl/fl^PTEN^fl/fl^H3.3K27M^mut^ mice, respectively. (F) Suvival comparison between p53^fl/fl^, p53^fl/fl^PTEN^fl/fl^, and p53^fl/fl^PTEN^fl/fl^H3.3K27M^mut^ mouse models.

To further evaluate the recapitulation of our GEM model to pediatric tumors in humans, we performed IHC analysis for various markers. Both pDMG models (Fig. 3A) and pHGG models (Fig. 3B) revealed similar characteristics to post-mortem patient samples,^1^ with noticeable cellular atypia, microvascular proliferation, and pseudopalisading necrosis in *de novo* tumors (Fig. 3A and B). Molecular characteristics of pDMG were present in both GEM models, with a cellular invasion of adjacent parenchyma, Ki67 positivity, Olig-2 expression, the presence of CD11b + cells, low number of CD3 + cell, and heterogeneous IL13RA2 expression (Fig. 3A and B).

In order to develop targeted immunotherapies using these models, we generated cell lines from our *de novo* tumors for subsequent *in vitro* experiments. Brain tissue from tumor-bearing animals was processed in a single-cell suspension as described in the Material and Methods, placed into culture, and maintained as neurospheres. The expression of PDGFB and IL13RA2 was confirmed by both midline and cortical tumors by FSC analysis (Fig. 3C). To assess tumorigenicity, we orthotopically implanted these cell lines into the midline or right cortex of 4–5 days-old Nestin-tva p53^fl/fl^ mice. Survival analysis displayed a median survival of 42 and 41 days for midline and cortical tumor-bearing, respectively (Fig. 3D). IHC analysis of these tumors displayed histological features similar to those of *de novo* tumors, with heterogeneous IL13RA2 expression, Olig-2 expression, infiltration of CD11b + cells, and a small number of CD3 + cells along with Ki67- positivity (Suppl. Figure 1A).

The genomic landscape of pHGG is characterized by recurrent mutations in TP53, PTEN, and histone H3 variant H3.3 (H3F3A)^29,30^. In addition to Nestin-tva p53^fl/fl^ mice, we induced PTEN deletion into tumors using Nestin-tva p53^fl/fl^PTEN^fl/fl^ mice and introduced the H3.3K27M mutation. Immunohistological evaluation of PTEN expression in both models confirmed deletion of PTEN in p53^fl/fl^ PTEN^fl/fl^, but not p53^fl/fl^ mice (Fig. 3E). Similarly, staining with an antibody detecting H3K27me3 revealed absence of staining in tumors with H3.3K27M mutation, with an exception for cells of tumor microenvironment or surrounding brain. Dual deletion of PTEN and p53 and dual deletion of PTEN and p53 with H3.3K27M generated significantly more aggressive tumors compared to p53 alone, with a median survival of mice of 31.0, 35.0, and 52.5 days in p53^fl/fl^ PTEN^fl/fl^ and p53^fl/fl^, respectively (Fig. 3F). No difference in survival was seen upon introduction of the H3.3K27M compared to PTEN and p53 dual deletion (Fig. 3F).

#### Response of de novo tumors to CAR T- cell therapy.

**Figure 4:**
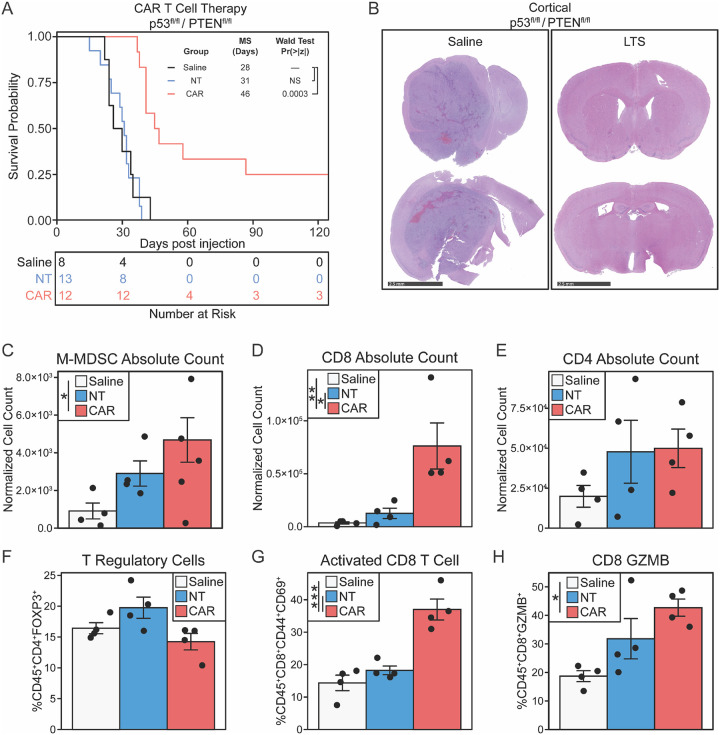
*De novo* PDGF-B/IL13RA2 overexpressing tumors respond to CAR T-cell therapy. (A) Survival analysis of mice with *de novo* cortical tumor of Nestin-Tva; p53^fl/fl^/ PTEN ^fl/fl^ mice treated with saline (n=8), non-transduced (NT) T-cells (n=13) or CAR T-cells (n=12), ***p<0.0001. (B) H&E stain of mouse brain from control (left panel) and CAR T-cell-treated (right panel) long-term surviving mice. (C-H) Flow cytometric analysis of tumor microenvironment of nestin-Tva; p53^fl/fl^/ PTEN ^fl/fl^ mice treated with saline (white), non-transduced (NT) T-cells (blue) or CAR T-cells (red).

To investigate the suitability of our GEM model of pHGG to targeted therapy, we generated murine IL13RA2-CAR T-cells, as described previously.^22^ The neurospheres of glioma cells established from cortical (Suppl. Figure 1B, C, D, and E) and midline tumors (Suppl. Figure 1F, G, H, and I) were labeled with CFSE, co-cultured with control non-transduced (NT) or CAR T-cells (CAR), and then characterized via flow cytometric analysis (gating strategy Suppl. Figure 2A and B). Both models demonstrated a significant response to CAR T-cells (Suppl. Figure 1C, Suppl. Figure 1G), with target-dependent activation of CD8+ (Suppl. Figure 1D, Suppl. Figure 1H) and CD4+ (Suppl. Figure 1E, Suppl. Figure 1I) CAR T-cells as judged by expression of CD25 and CD69 markers. Following confirmation of functionality, we tested the response to targeted therapy *in vivo* by treating cortical tumor-bearing mice with CAR T-cell therapy. Intracranial delivery of CAR T-cells significantly improved the survival (MS: 46 days) compared to both saline (MS: 28 days), and NT-treated animals (MS: 31 days) (Wald p-value < 0.001) (Fig. 4A). Furthermore, 25% of mice (3 out of 12) treated with CAR T-cells survived long-term (LTS) (Fig. 4A), and histological analysis confirmed absence of tumor in these mice (Fig. 4B). To understand how targeted therapy effects TME cellular composition, we again treated cortical tumor-bearing mice (Suppl Fig. 1A) with CAR T cell therapy, harvested and processed the brains of CAR T-cell (CAR), non-transduced T-cell (NT), and saline treated animals into single cell suspensions, and performed multicolor flow cytometric analyses (gating strategy Suppl. Figure 2C). We observed significantly increased frequency of monocytic MDSC (M-MDSC; CD45^+^CD11b^+^Ly6C^+^Ly6G^lO^) (Fig. 4C) and frequency of CD8 T-cells (Fig. 4D), but not CD4 (Fig. 4E), in the brains of CAR T cell treated animals comapred to saline. We observed no change in the frequency in T regulatory cells (CD45^+^CD4^+^FOXP3^+^) (Fig. 4F). Furthermore, we also observed increased frequency of activated (CD45^+^CD8^+^CD44^+^CD69^+^) (Fig. 4G) and cytotoxic (CD45^+^CD8^+^GZMB^+^) (Fig. 4H) CD8 T-cells in CAR treated animals compared to saline. Together, these experiments indicate that *de novo* established aggressive glioma tumors expressing IL13RA2 are responsive to CAR T-cell therapy, and thus, are suitable for investigating responses and resistance of aggressive pediatric diffuse gliomas to targeted therapeutics in mice with fully competent immune system.

## DISCUSSION

Amongst many factors, the efficacy of targeted immunotherapy depends on the targeted antigen’s validity. IL13RA2 is an attractive TAA present in several CNS tumors and has been validated as an immunotherapeutic target in adult GBM^31,32^. To better understand IL13RA2 as a potential immunotherapeutic target in the context of pHGG, we first characterized its expression in datasets of patient tissue samples. We found that IL13RA2 is expressed at higher levels in pHGG, notably with around 45% of DIPG samples expressing IL13RA2, an observation in agreement with other reports^8,13^. These preliminary results suggest that IL13RA2 has the potential to be used as an immunotherapeutic target for pDMG and pHGG.

A significant obstacle to developing targeted immunotherapy for pDMG and pHGG is the lack of an immunocompetent model expressing human antigens. We used the RCAS-Tva system to overcome this limitation and induce *de novo* tumorigenesis in immunocompetent mice. In doing so, we successfully generated pDMG and pHGG that express IL13RA2, and the addition of IL13RA2 to PDGFB-driven gliomas did not significantly impact the survival compared to the previously described PDGFB-driven tumor model of pediatric diffuse midline glioma^33^. Moreover, *de novo* tumors with a dual p53/PTEN deletion acquired a more aggressive phenotype than tumors with p53 deletion alone. Finally, our generated tumors recapitulated the histological and molecular hallmarks seen in patient samples, such as cellular atypia, microvascular proliferation, pseudo palisading necrosis, and invasion into surrounding brain tissue.

Tumor-derived cell lines derived express IL13RA2 and reliably developed tumors when implanted orthotopically in the murine brain. Consistent with human pDMG and pHGG^34,35,25^, both *de novo* and orthotopically generated tumors displayed a heterogeneous expression of IL13RA2 with limited infiltration CD3 T-cells and a moderate CD11b myeloid cells in the TME. Our analysis of tumor-infiltrating T-cells and myeloid cells following CAR T-cell therapy revealed increased frequency of CD8 T-cells and monocytic MDSCs (M-MDSCs). M-MDSCs are normally recruited to sites of inflammation to help modulate immune responses and prevent excessive tissue damage. Once there, they can actively suppress T-cell response by expressing high levels of immunosuppressive molecules such as ARG1 and iNOS. Given the increased presence of tumor-infiltrating activated/cytotoxic CD8 T-cells following CAR T-cell treatment, a role of M-MDSC in supporting or dampening the tumor responses to CAR T-cells needs to be further investigated. Future studies employing single-cell RNA sequencing and multiplex immunofluorescence to characterize the TME will help to understand the changes taking place upon the introduction of immunotherapy and how these changes play a role in immunotherapeutic efficacy.

Multiple recurrent mutations characterize the genomic landscape of pHGG. TP53 mutations, affecting the well-studied tumor suppressor protein p53, occur in over 40% of patients^36^. PTEN mutations are present in 10–20% of pHGG cases^29,30^. The most common mutation is in H3F3A, the gene encoding histone H3 variant H3.3. This mutation involves a lysine-to-methionine substitution at position 27 (H3.3K27M) and occurs in 70–80% of cases^36,37^. Furthermore, a strong positive correlation between IL13RA2 expression and H3.3K27M mutation has been found in pHGG and pDMG^32,38^. In this study, we successfully used the RCAS-Tva model to generate H3.3K27M mutant tumors lacking p53 and PTEN that express IL13RA2. Furthermore, we demonstrate that *de novo* IL13RA2-expressing tumors respond to CAR T-cell therapy, with some animals surviving long term. The strength of the GEM model is its versatility in incorporating molecular alterations, and this work serves as a proof of concept that relevant human antigens can be readily incorporated into GEM models and may serve as useful preclinical tools.

Clinical studies using dual and multispecific targeting approaches have emerged in recent years. There is currently one clinical study using B7-H3-IL13RA2-CAR T cells in pediatric diffuse gliomas (NCT06221553); Another clinical study featuring a quad-specific B7H3-HER2-EGFR-IL13RA2 CAR T-cell in pediatric diffuse gliomas (NCT05768880). By incorporating additional human tumor-associated antigens (e.g., B7-H3, EGFRvIII, GD2, and HER2), this model could strengthen multi-targeting approaches for pediatric diffuse gliomas by enabling preclinical development in an immunocompetent host.

## CONCLUSION

We developed a fully immunocompetent, genetically engineered mouse model (GEMM) for pDMG and pHGG that incorporates the glioma-associated antigen, IL13RA2 and demonstrated the suitability of the model for evaluation of targeted therapy such as CAR T cells. We envision that this model will permit preclinical assessment of several human antigen-targeting immunotherapeutics, including CAR-T cells, bi- or multispecific T and NK cells engaging antibodies, and antibody-drug conjugates therapies within an immunocompetent host. After establishing the potential anti-tumor effect of each of these therapies, combinatorial approaches with concurrent radiation, chemotherapy, or targeted therapies, or in conjunction with other immunotherapeutic approaches, such as checkpoint inhibitors or other antibodies, will be considered. A robust evaluation of such strategies will allow for potential clinical translation to improve the outcome for children impacted by these devastating tumors.

## Abbreviations

ARG1: Arginase 1
BCA: Bicinchoninic Acid
CAR T-cells: Chimeric Antigen Receptor-Modified T-Cells
CNS: Central Nervous System
DMEM: Dulbecco’s Modified Eagle’s Medium
DMG: Diffuse Midline Gliomas
DIPG: Diffuse Intrinsic Pontine Glioma
FBS: Fetal Bovine Serum
FSC: Flow Cytometry
GAPDH: Glyceraldehyde 3-Phosphate Dehydrogenase
GEMM: Genetically Engineered Mouse Model
GZMB: Granzyme B
H3F3A: Histone H3 Variant H3.3
H&E: Hematoxylin and Eosin
HSD: Honest Significant Difference
IACUC: Institutional Animal Care and Use Committee
IL13RA2: Interleukin 13 Receptor Alpha 2
IHC: Immunohistochemistry
iNOS: Inducible Nitric Oxide Synthase
LTS: Long-Term Survival
M-MDSCs: Monocytic Myeloid-Derived Supressor Cells
mPER: Mammalian Protein Extraction Reagent
NFDM: Non-Fat Dry Milk
PBS: Phosphate-Buffered Saline
PFA: Paraformaldehyde
pDMG: Pediatric Diffuse Midline Gliomas
pHGG: Pediatric High-Grade Glioma
T:E: Target-to-Effector
